# The factor validity of the Western Ontario Rotator Cuff Index

**DOI:** 10.1186/1471-2474-6-22

**Published:** 2005-05-04

**Authors:** Jean Wessel, Helen Razmjou, Yasmin Mewa, Richard Holtby

**Affiliations:** 1 School of Rehabilitation Science, McMaster University, Hamilton, Canada; 2 Department of Rehabilitation, University of Toronto, Orthopaedic and Arthritic Institute, Sunnybrook & Women's College Health Sciences Centre, Toronto, Canada; 3 Student in Medical Programme, University College Cork, Cork, Ireland; 4 Department of Surgery, University of Toronto, Orthopaedic and Arthritic Institute, Sunnybrook & Women's College Health Sciences Centre, Toronto, Canada

## Abstract

**Background:**

The Western Ontario Rotator Cuff Index (WORC) is a self-report questionnaire developed specifically to evaluate disability in persons with pathology of the rotator cuff of the shoulder. The authors created items in 5 categories based on a model of quality of life, but never validated this structure. The purpose of this study was to examine the validity of the original 5-domain model of the WORC by performing factor analysis.

**Methods:**

Three hundred twenty nine subjects (age, mean: 52, SD: 12) were tested prior to undergoing surgery for rotator cuff pathologies. They completed the WORC, a self-report questionnaire, which has 21 items on the effect of the rotator cuff problem on symptoms, activities and emotions. Statistical calculations included correlations between items, Cronbach's alpha of the total scale and subscales, and principal component factor analysis with oblique rotation.

**Results:**

Correlations ranged from .09 to .70 between all the items, from .29 to .70 between items within a subscale, and from .53 to .72 between subscale scores. Cronbach's alpha was .93 for the total scale, and .72 to .82 for the subscales. The factor analysis produced 3 factors that explained 57% of the variance. The first factor included symptoms and emotional items, the second included strength items and the third included daily activities.

**Conclusion:**

The results of this study did not support the 5-domain model of the WORC.

## Background

The Western Ontario Rotator Cuff Index is a recent self-report questionnaire that was designed to measure "health-related quality of life" in persons with injuries and conditions of the rotator cuff of the shoulder. Kirkley et al [1] felt the measure should represent the impact of the condition on health as defined by the World Health Organization – "a state of complete physical, mental and social well-being". They, therefore, included items in 5 domains in the questionnaire: 1) pain and physical symptoms, 2) sports and recreation, 3) work, 4) lifestyle, and 5) emotions. The authors followed a systematic, clinimetric method of generating and reducing the items. This resulted in 21 items that respondents answered on visual analogue scales (VAS) with anchors such as no pain/difficulty and extreme pain/difficulty.

Items for the WORC were derived from published health status scales, functional measures of the shoulder, discussions with healthcare professionals, and interviews with 30 patients from a registry of 150 with rotator cuff pathology. Both professionals and patients were asked to identify ways in which the shoulder condition affected quality of life in general, and the 5 domains in particular. The 30 patients interviewed included males and females, aged 30–76, with different degrees of rotator cuff pathology from tendinitis to massive tears.

An original list of 321 items was reduced to 76 by the investigators eliminating duplicated, incomprehensible or ambiguous items. A random selection of 100 patients from the same registry were then asked to indicate whether they experienced each of the items, and to rate the importance of the symptom/disability to their overall shoulder functioning. A frequency importance product was calculated for each item and the 50 items with the highest values were correlated with each other. For every pair of items with coefficients greater than 0.6, one of the items was eliminated, resulting in the final 21 questions. It is not clear whether this criterion applied to items across domains because the only example provided included 3 items from the same domain.

In that same paper [1], the authors reported an ICC for reliability of .96 when they tested subjects over a 2-week period and omitted those who reported any change on a global rating scale. The ICCs for the subscales ranged from .54 (4-item work) to .91 (6-item physical symptoms).

Construct validity has been tested by the original authors [1] and others [2,3] by examining the correlation of the WORC with other shoulder measures (Constant, American Shoulder and Elbow Surgeons Standardized Shoulder Assessment Form [ASES], Disabilities of the Arm, Shoulder and Hand [DASH], University of California Los Angeles [UCLA], Simple Shoulder Test [SST]). The correlations of the WORC total score with the other instruments have ranged from .48 to .91, with generally higher correlations with instruments that have disability items similar to those in the WORC (see Table 1). The correlations of the change scores were in a similar range (.44 to .85).

**Table 1:** Correlations of the WORC with other shoulder measures

Article	Scores	Constant	ASES	DASH	UCLA	SST
Kirley et al [1]	Cross-sectional	.57		.63	.48	
	Change	.44	.76	.66	.72	
Holtby & Razjmou [3]	Cross-sectional	.61–.66	.73			
	Change	.70–.77	.85			
Getahun et al [2]	Cross-sectional			.88		.91

Two studies [3,4] have examined the responsiveness of the WORC and other shoulder measures by calculating the standardized response mean (SRM) in patients who have been measured before and after surgery (see Table 2). It should be noted that the SRM of the WORC was not noticeably different from the comparative measures (Constant, SST and DASH) in the same study. Holtby and Razmjou [3] had lower overall SRMs than MacDermid et al [4] who included only the responders in their calculations. MacDermid et al [4] also reported the SRM for the subscales of the WORC. The values ranged from 1.2 for the work subscale to 1.8 for the lifestyle subscale.

**Table 2:** Standardized response means for the WORC and other shoulder measures

	WORC	Constant (absolute)	Constant (relative)	ASES	DASH	SST
Holtby & Razjmou [3]	1.3	1.4	1.3	.9		
MacDermid et al [4]	2.1				1.8	2.0

When Kirkley et al [1] developed the WORC, they argued for the use of "disease-specific" measures to evaluate orthopedic treatment because they are more responsive than global health measures. However, they set out to develop, not only measures specific to the shoulder, but instruments specific to conditions of the shoulder. Now there exist Western Ontario tools for the measurement of disability in shoulder instability (WOSI) [5] osteoarthritis [6] and rotator cuff conditions [1].

The results provided above, however, suggest that generic measures of the shoulder may perform as well as condition-specific measures. The WORC was highly correlated with both the DASH and the SST [2], and had a standardized response mean similar to these two instruments [4]. Therefore, it may not be necessary to have a tool that is specific to a particular condition in the shoulder. Moreover, the WORC is more time consuming to complete and to score, and may not be as attractive as the other scales for use in a clinical setting.

One advantage of the WORC may be its comprehensiveness. It was designed to tap 5 domains of health and may provide information that is unavailable in the other measures. However, the subscales have not been studied in detail, nor has there ever been a confirmation that the WORC items fall into the 5 domains. The purpose of the present study was to examine the validity of the original 5-domain model of the WORC by performing factor analysis.

## Methods

### Subjects

The data were drawn from a database that included all patients who were to undergo arthroscopic acromioplasty for surgical management of impingement or rotator cuff pathology of the shoulder (Table 3) in a tertiary level hospital in Toronto, Canada between October 2000 and July 2004. Complete data were available on 329 (196 males, 133 females) out of a total of 334 patients. All patients subsequently underwent arthroscopic acromioplasty with or without rotator cuff repair. A number of patients had superior labral anterior and posterior (SLAP) lesions that required surgical repair.

### Design

The data of this study were prospectively collected. All patients were sent a number of questionnaires that included the WORC, 3–4 weeks before surgery via mail. Just prior to surgery, the patients were then seen by a physical therapist who performed some physical tests (not reported in this study), and checked that the questionnaires were completed. The data extracted for this study included demographics (Table 3) and the scores on each of the individual items of the WORC questionnaire.

**Table 3:** Subject characteristics

**Variable**	Mean or Frequency*	**Percent**
Age (years)	52.2 (23–81)	
Gender (female, male)	133, 196	40, 60
Duration of symptoms		
≤ 1 year	109	33
1–2 years	151	46
>2–5 years	3	1
>5 years	53	16
missing data	13	4
Dominant side		
Right	294	89
Left	26	8
Ambidextrous:	9	3
Affected side		
Right	202	61
Left	127	39
General health status		
Good	184	56
Diabetes	27	8
Chronic illness	60	18
Other	47	14
Missing data	11	3
Surgery		
Acromioplasty	329	100
Resection of distal clavicle	107	32
Rotator cuff repair	96	29
SLAP repair	32	10

*Mean and range of age are provided. The remaining values are frequencies

### WORC measure

As indicated previously, the WORC is a 21-item questionnaire examining the impact of rotator cuff pathology on "quality of life". Subjects answer each question on a 100 mm visual analogue scale and the higher numbers indicate worse pain or difficulty. The questions in each of the theoretical domains are presented together. The WORC total is obtained by adding the scores on all the items. The subscale scores are totals of the item scores in that domain.

The WORC questionnaire has been published in full [1]. However the 1998 copyright version obtained from the authors and used in this study varies slightly from the published version. The minor differences are noted in Table 4.

**Table 4:** Differences between copyright and published versions of WORC

Item	Published version [1]	1998 copyright version used in present study
PS 4	How much stiffness do you...	How much stiffness *or lack of range of motion *do you...
PS 5	How much *do you experience *clicking...	How much *are you bothered by *clicking...
PS 6	How much discomfort do you experience in your neck...	How much discomfort do you experience in *the muscles of *your neck...
	SR 8	SR 9
	SR 9	SR 10
	SR 10	SR 8
W 12	How much...above your head	How much...above your shoulder
W 14	How much...objects *from the ground *or below shoulder level	How much...objects *at or *below shoulder level
W 21	How worried...occupation *or work*?	How worried...occupation?

PS physical symptoms

SR sports and recreation

W work

### Data analyses

Descriptive statistics were calculated for the 21 items, for the subscale scores and for the total WORC score. Correlations between the items and between the subscales were examined with Pearson Product Moment Correlations. A Cronbach's alpha was calculated to determine the internal consistency of the total score and the subscale scores.

Principal component analysis was the extraction method used for the factor analysis. Only factors with eigenvalues greater than 1 were considered. The Kaiser-Meyer-Olkin Measure and Bartlett's Test of Sphericity were performed to determine whether the data were suitable for factor analysis. [7] Because all the subscales were correlated, an oblique rotation method (SPSS direct oblimin option, SPSS version 11.0.1, SPSS Inc) was used. An item was considered to be loaded on a factor if its pattern matrix coefficient was .5 or greater. We also noted those items that loaded between .4 and .5 but had no higher loading on another factor.

## Results

The descriptive statistics, alpha coefficients and inter-item correlations are outlined in Table 5. The correlations between items ranged from .09 to .70, with the lowest correlations being between the emotion items and two of the sports/recreation items. The correlations between items within a subscale varied between .29 and .70. The correlation between subscale scores varied between .53 and .72. Internal consistency of the subscales was .72 to .82 (Cronbach's alpha). The Cronbach's alpha for the total scale was .93.

**Table 5:** Descriptive statistics for WORC item and subscale scores

Item*	Item mean	Item SD	Subscale mean (SD)	Cronbach's alpha	Range of inter-item correlations within subscale
PS1	66	25.5	382 (121.7)	.81	.32 – .60
PS2	64	27.3			
PS3	71	24.9			
PS4	68	24.9			
PS5	57	33.7			
PS6	56	32.2			

SR7	69	28.5	302 (81.8)	.72	.29 – .55
SR8	86	21.6			
SR9	83	27.6			
SR10	64	32.2			

W11	65	25.7	296 (78.5)	.78	.38 – .64
W12	85	19.1			
W13	77	24.4			
W14	69	30.8			

LS15	68	29.0	255 (96.3)	.82	.42 – .68
LS16	56	34.3			
LS17	75	27.3			
LS18	55	28.4			

E19	77	27.1	196 (84.8)	.80	.52 – .70
E20	58	34.6			
E21	62	37.7			

PS physical symptoms

SR sports and recreation

W work

* See Table 6 for explanation of items

The data met the criteria for factor analysis. As can be seen from Table 6, the factor analysis revealed 3 factors that explained 57% of the variance. The factors converged in 19 iterations. Factor 1 included all the emotional items and some symptoms not related to specific tasks (shoulder clicking, neck discomfort, and affect on fitness). Three additional items loaded between .4 and .5. They were all questions about pain. Two of the sports items (ability to throw hard/far, and difficulty with push-ups) loaded on factor 2, with the weakness item loading between .4 and .5. The third factor included several items that asked about difficulty performing specific activities. The Cronbach's alpha values for the three factors were: .87 (9 items), .67 (3 items) and .89 (8 items) respectively.

**Table 6:** Pattern matrix following oblique rotation [listed by items loading on a given factor]

		Factors		
		
		1	2	3
	Item	Emotions & symptoms	Disability – strength activities	Disability – daily activities

PS5	Shoulder clicking, grinding, crunching	**.64**	.15	<.01
PS6	Neck discomfort	**.52**	<.01	-.16
SR7	Affect fitness level	**.58**	.45	<.01
E19	How much frustration	**.73**	<.01	<.01
E20	How depressed	**.77**	-.13	-.11
E21	How worried about effect on occupation	**.81**	<.01	<.01
PS1	Sharp pain	*.47*	<.01	-.29
PS2	Constant, nagging pain	*.49*	*<.01*	-.34
LS15	Difficulty sleeping	*.45*	-.21	*-.45*

SR8	Difficulty with push-ups	.14	**.85**	.11
SR9	Affect ability to throw	-.14	**.71**	-.17
PS3	How much weakness	.23	*.41*	-.28

PS4	How much stiffness	<.01	.25	**-.56**
SR10	Difficulty with contact with shoulder	.27	.12	**-.53**
W11	Difficulty with daily house/yard activities	.30	.15	**-.56**
W12	Difficulty working above shoulder	<.01	.33	**-.55**
W14	Difficulty lifting heavy objects	<.01	<.01	**-.63**
LS16	Difficulty styling hair	<.01	-.14	**-.88**
LS18	Difficulty dressing/undressing	<.01	<.01	**-.82**
LS17	Difficulty roughhousing	.20	.31	*-.46*

W13	How much use of uninvolved arm	<.01	.36	-.38

Factor loadings > 0.5 are in bold.

Factor loadings between 0.4 and 0.5 are in italics if that item did not load higher on another factor

E emotions

LS lifestyle

PS physical symptoms

SR sports and recreation

W work

To see the factor loadings with items listed by the domains of the original scale, see additional file 1: Pattern Matrix by domains.doc.

## Discussion

The main purpose of this study was to determine whether the WORC items fell into 5 domains as proposed by the creators of the scale. Although some of the items grouped together as hypothesized, the factor analysis did not support the 5-domain construct of the WORC.

The factor analysis revealed 3 factors, not 5. The 3 factors appear to be: 1) symptoms and emotions, 2) strenuous shoulder tasks, and 3) difficulty with daily tasks. Based on the groupings, it appears that symptoms of pain are associated with emotions, and lack of range of motion or stiffness with difficulty with daily activities. The symptom of "weakness' was associated with two very specific shoulder tasks – throwing hard and push-ups. Based on the mean values for these two items (S8, S9), they were likely the most difficult tasks as well. Thus it is not surprising that "weakness" was associated with difficulty with these activities.

Although factor analysis has not been previously performed on the WORC, other authors have reported a mix of symptoms, disability and social/emotional items within factors derived from other shoulder measures. Veehof et al [8] noted that all 30 items of the DASH loaded positively on the first factor following principal component factor analysis. Only 3 loaded less than .50. The DASH has questions on physical function, symptoms and social/role function. Similarly, Roddey et al [9] reported that both the pain and disability items of the SPADI loaded on one factor (.613 to .905). On the other hand, two factors were derived from the Simple Shoulder Test (SST) [9], which was designed to measure one construct, functional ability in activities of daily living. All of these results suggest that patients with shoulder problems may not differentiate disability and symptoms, and that such theoretical groupings of items are not appropriate.

This lack of separation of pain and disability has been seen in measures of the lower limb as well. Kennedy et al [10] found that the items of the Western Ontario and McMaster Osteoarthritis Index (WOMAC) factored out on type of activity rather than pain or difficulty. The authors [10] felt their results might be due to the similarity of the questions in the two domains. For example, 'pain with sitting or lying' is in the pain subscale, and 'difficulty with lying in bed' and 'difficulty with sitting' are both in the physical function subscale. There is no such duplication in the WORC items, and yet, in the present study, there was at least one symptom question, and one "difficulty" question in each factor. Thus, it may be that individuals do not inherently separate symptoms and functional ability in musculoskeletal conditions, no matter how the questions are worded.

In their systematic review of shoulder measures, Bot et al [11] considered a measure to have good internal consistency when its structure was explored by factor analysis, and Cronbach's alpha for each separate factor was .70 to .90. Two of the three subscales derived from the factor analysis met this criterion. The middle factor/subscale, with only 3 items, did not meet the .70 criterion. However, the Cronbach's alpha increased to .70 when the weakness item, which loaded only .41, was removed. The other two factor/subscales had alpha coefficients higher than the original subscales.

The WORC was originally developed and tested on a heterogeneous group of patients that likely had a wider range of disease severity than the pre-surgical patients used in the present study. Because Kirkley et al [1] did not present any descriptive statistics for the total or subscale scores of the WORC, the actual range of disability of the subjects in the two studies can not be directly compared. Even so, it is possible, that the results might have been closer to the 5-domain model proposed by Kirkley et al [1] if the subjects were similar in the two studies. However, one would expect a robust measure to have similar properties when used on all types of patients for which it was intended. Additional research should be conducted to confirm the subscales found in the present study, to examine their properties and determine the value of their use in the clinical or research setting.

## Conclusion

The results of this study indicate that the WORC has 3 factors, which explain 57% of the variance. All factors include both 'function' and 'symptom' questions. The three items from the original emotional scale were the only ones that grouped together, but that factor also included items from 3 other subscales. The results of this factorial analysis do not support the 5-domain structure proposed by the creators of the WORC. Based on the results of the present study and on previous work conducted on the WORC, there does not appear to be a significant advantage to using this condition-specific questionnaire over some other well-established measures for the shoulder.

## Competing interests

The author(s) declare that they have no competing interests.

## Authors' contributions

JW proposed the study, developed the research protocol, and wrote the first draft of the paper. HR was responsible for subject selection, data collection and management of the database. YM was involved in the review of the literature, data input and initial analysis of data. RH was involved in patient recruitment and providing clinical and surgical diagnosis. All authors were involved in the preparation of the manuscript, and read and approved the final version.

## Pre-publication history

The pre-publication history for this paper can be accessed here:



## Supplementary Material

Additional File 1 Word file showing the factor loadings grouped by the theoretical domains Click here for file
