# Supplementary material for: The factor validity of the Western Ontario Rotator Cuff Index
Source: BMC Musculoskelet Disord. 2005 May 4;6:22. doi: 10.1186/1471-2474-6-22 (PMC1112598; doi:10.1186/1471-2474-6-22)
Supplement: Additional File 1 — Word file showing the factor loadings grouped by the theoretical domains [file 1471-2474-6-22-S1.doc]

**Pattern matrix following oblique rotation [listed by theoretical domains]**

|  | Factors | | |
| --- | --- | --- | --- |
| Item | 1 | 2 | 3 |
| Physical symptoms 1 | *.47* | <.01 | -.29 |
| 2 | *.49* | *<.01* | -.34 |
| 3 | .23 | *.41* | -.28 |
| 4 | <.01 | .25 | **-.56** |
| 5 | **.64** | .15 | <.01 |
| 6 | **.52** | <.01 | -.16 |
| Sports/recreation 7 | **.58** | .45 | <.01 |
| 8 | .14 | **.85** | .11 |
| 9 | -.14 | **.71** | -.17 |
| 10 | .27 | .12 | **-.53** |
| Work 11 | .30 | .15 | **-.56** |
| 12 | <.01 | .39 | **-.55** |
| 13 | <.01 | .36 | -.38 |
| 14 | <.01 | <.01 | **-.63** |
| Lifestyle 15 | *.45* | -.21 | *-.45* |
| 16 | <.01 | -.14 | **-.88** |
| 17 | .20 | .31 | *-.46* |
| 18 | <.01 | <.01 | **-.82** |
| Emotion 19 | **.73** | <.01 | <-.01 |
| 20 | **.77** | -.13 | -.11 |
| 21 | **.81** | <.01 | <.01 |

Factor loadings > 0.5 are in bold.

Factor loadings between 0.4 and 0.5 are in italics if that item did not load higher on another factor
